# Research Status of the Orphan G Protein Coupled Receptor 158 and Future Perspectives

**DOI:** 10.3390/cells11081334

**Published:** 2022-04-14

**Authors:** Xianan Fu, Shoupeng Wei, Tao Wang, Hengxin Fan, Ying Zhang, Clive Da Costa, Sebastian Brandner, Guang Yang, Yihang Pan, Yulong He, Ningning Li

**Affiliations:** 1 Tomas Lindhal Nobel Laureate Laboratory, The Seventh Affiliated Hospital of Sun Yat-sen University (SYSU), No.628, Zhenyuan Rd., Guangming Dist., Shenzhen 518107, China; fuxn@mail.sysu.edu.cn (X.F.); weishoupeng@sysush.com (S.W.); wangtao@sysush.com (T.W.); fanhx0308@hotmail.com (H.F.); zhangying@sysush.com (Y.Z.); panyihang@sysush.com (Y.P.); 2 The Francis Crick Institute, 1 Midland Road, London NW1 1AT, UK; clive.dacosta@crick.ac.uk; 3 Department of Neurodegenerative Disease, Institute of Neurology, University College London, Queen Square, London WC1N 3BG, UK; s.brandner@ucl.ac.uk; 4 Department of Burn and Plastic Surgery, Institute of Translational Medicine, Shenzhen Second People’s Hospital, The First Affiliated Hospital of Shenzhen University, Health Science Center, Shenzhen 518039, China; yakoaka@foxmail.com; 5 Center for Digestive Disease, The Seventh Affiliated Hospital of Sun Yat-sen University (SYSU), No.628, Zhenyuan Rd., Guangming Dist., Shenzhen 518107, China; 6 China-UK Institute for Frontier Science, Shenzhen 518107, China

**Keywords:** GPCR, GPR158, cancer, psychological disorders

## Abstract

G-protein-coupled receptors (GPCRs) remain one of the most successful targets for therapeutic drugs approved by the US Food and Drug Administration (FDA). Many novel orphan GPCRs have been identified by human genome sequencing and considered as putative targets for refractory diseases. Of note, a series of studies have been carried out involving GPCR 158 (or GPR158) since its identification in 2005, predominantly focusing on the characterization of its roles in the progression of cancer and mental illness. However, advances towards an in-depth understanding of the biological mechanism(s) involved for clinical application of GPR158 are lacking. In this paper, we clarify the origin of the GPR158 evolution in different species and summarize the relationship between GPR158 and different diseases towards potential drug target identification, through an analysis of the sequences and substructures of GPR158. Further, we discuss how recent studies set about unraveling the fundamental features and principles, followed by future perspectives and thoughts, which may lead to prospective therapies involving GPR158.

## 1. Introduction

In this review, we address the current status of research on GPR158, specifically its roles in cancer and mental illness.

**Figure 1:**
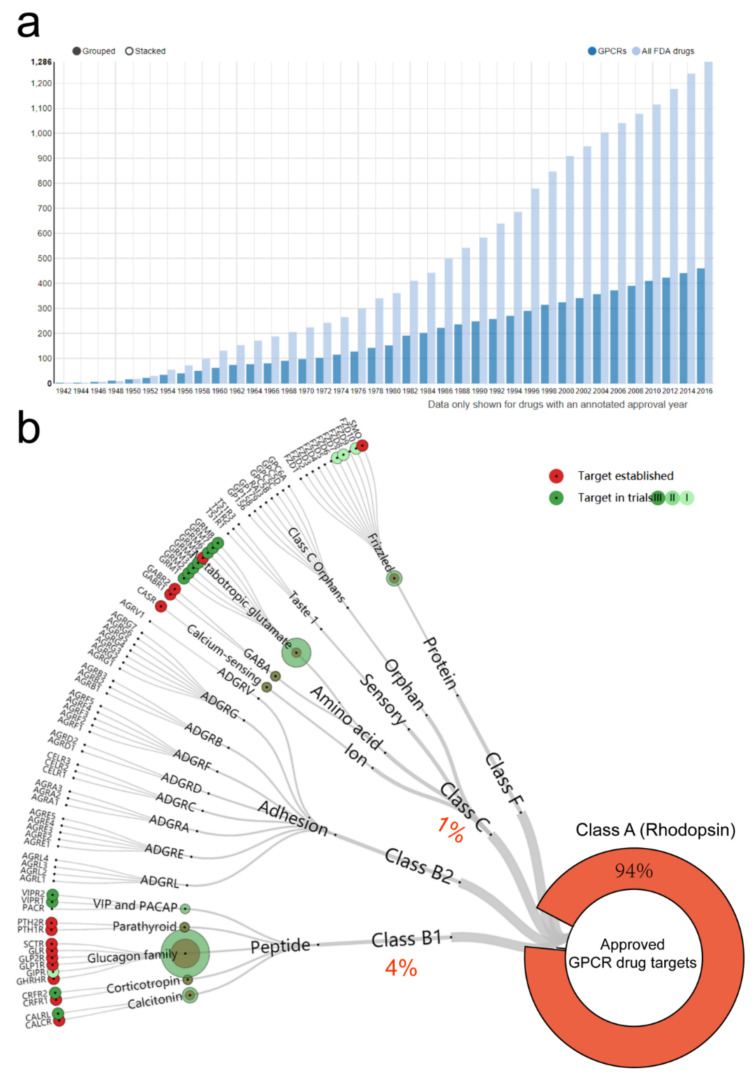
Research status of GPCR drug targets. (**a**) GPCRs share ~30% of FDA-approved drugs. Data are only shown for drugs with an annotated approval year and downloaded from https://www.gpcrdb.org/drugs/drugstatistics (last accessed on 12 April 2022). (**b**) The interactive tree that includes the number of agents for each target is downloaded and modified from http://www.gpcrdb.org/drugs/drugmapping (last accessed on 12 April 2022) [1,8,9]. Approved drugs (red) and phase I–III (green). The sizes of the circles represent the number of agents.

GPCRs have been of long-standing interest as pharmacological targets and represent ~30% of FDA-approved drugs as they evidence druggable sites at the cell surface and are the largest protein family in the human proteome with more than 800 members (Figure 1a) [1,2]. The GPCR superfamily can be broadly categorized into the following classes: A, B1, B2, C and F, based on evolutionary homology and receptor families with common physiological ligands, which are strikingly diverse, spanning ions, small molecular molecules, lipids, peptides and proteins. Firstly, class A GPCRs are rhodopsin-like receptors, engaging more than 700 in humans as the largest cluster [3]. Secondly, class B1 GPCRs are secretin receptor-like receptors, having a large extracellular N-terminal domain, a high-affinity peptide-binding site, and a lower affinity site localized between the heptahelical helices [4]. Thirdly, class C GPRCs are obligatory dimers (reviewed in [5]), with 15 types of orphan receptors in humans. The dimers in most cases are stabilized by covalent sulfhydryl bonds, though the separated Venus Flytrap Domain (VFD) can form dimers without S–S bonds, as their bacterial ancestors. Lastly, class B2 GPCRs and F GPCRs contain a large extracellular domain (ECD) and can be activated via complicated mechanisms that are relatively poorly understood [6,7]. To date, therapeutics have predominantly targeted class A and B GPCRs (Figure 1b) [8]. However, the function and activation mechanism of other GPCR classes, such as class C, remains elusive. In this review, we focus on the novel class C Orphan GPCR-GPR158 and discuss its fundamental features.

GPCRs are able to sense environmental information at the earliest stage by binding extracellular ligands, and then intracellularly relay these signals by interacting with G proteins. Orphan GPCRs refer to the receptors for which the endogenous ligands have not yet been discovered. Currently, several orphan receptors are attractive drug targets with important roles in physiology and disease [10], such as GPR119 for diabetes [11], LGR5 for gastrointestinal diseases [12], GPR35 for allergic inflammation [13], GPR84 for ulcerative colitis [14], and GPR50 for bipolar affective disorder [15]. Present data indicate that, despite the endogenous ligands that remain unidentified or the underlying signaling pathways that are uncomprehended, drug target evaluation is worth pursuing simultaneously. Among orphan GPCRs, GPR158 is of particular interest as a novel GPCR, as it has been shown to play a critical role in the etiology of cancers and mental illnesses, e.g., prostate cancer [16], glioma [17] and depression [18,19], though the properties of GPR158 are yet to be fully elucidated.

## 2. Discovery of GPR158

Bjarnadottir, et al. assembled GPR158 in 2005 and clustered vertebrate glutamate GPCRs into four phylogenetic groups (Group I: V2R, TAS1R, GPRC6A and CASR; Group II: GRM; Group III: GABA, GPR158 and GPR158L; Group IV: GPRC5) [20]. Furthermore, they clarified that GPR158 notably appeared in mammals, but not in fish, although there is evidence to show its expression in killifish [21]. The GPR158 gene is only present in vertebrates and highly conserved in chimpanzees, monkeys, dogs, cows, mice, rats, chickens, zebrafish and frogs. Although GPR158 was assembled in *D. melanogaster*, it did not obtain hits upon reverse position through a specific BLAST (RPS-BLAST) search [20]. GPR158 has a paralogue named GPR179. GPR179-encoded protein has an EGF-like calcium-binding domain and a seven-transmembrane domain (7TM) in the N-terminal region. Mutations in the *Gpr179* gene are associated with congenital stationary night blindness [22]. In the NCBI gene annotation, the EGF-like calcium-binding domain was only in lower vertebrates, though the physiological function of the domain awaits further investigation [23]. The evolutionary history of GPR158 and GPR179 is a subject that needs to be studied further.

In later functional research, the Martemyanov group first identified that GPR158 controlled the localization and activity of regulator of G protein signaling (RGS) 7 and Gβ protein [24]. RGS7, as well as RGS6, RGS9 and RGS11, belongs to members of the R7 subfamily of RGS proteins that are highly expressed in neurons and play a role in multiple physiological processes [25]. GPR158 binding with RGS7 could negatively modulate Gi/o signaling [26]. G proteins are usually composed of α, β and γ subunits, and are divided into four families according to their Gα subunit composition: Gs/olf, Gi/o, Gq/11 and G12/13 [27]. In response to extracellular stimuli, GPCRs transduce signals downwards through G proteins, while each G protein activates distinct signaling pathways that enable divergent physiological processes [27]. The *Fini* laboratory reported that glucocorticoid (GC) treatment increases GPR158 mRNA and protein, and stimulates cell proliferation [28]. Recently, several research groups found that GPR158 could interact with several members of the heterogeneous family of cell-surface and secreted heparan sulfate proteoglycans (HSPGs) and osteocalcin (OCN) [29,30,31]. However, the functions of GPR158 are still largely unknown, although studies indicate an emerging key role in the nervous system and in cancer.

## 3. Structure of GPR158

**Figure 2:**
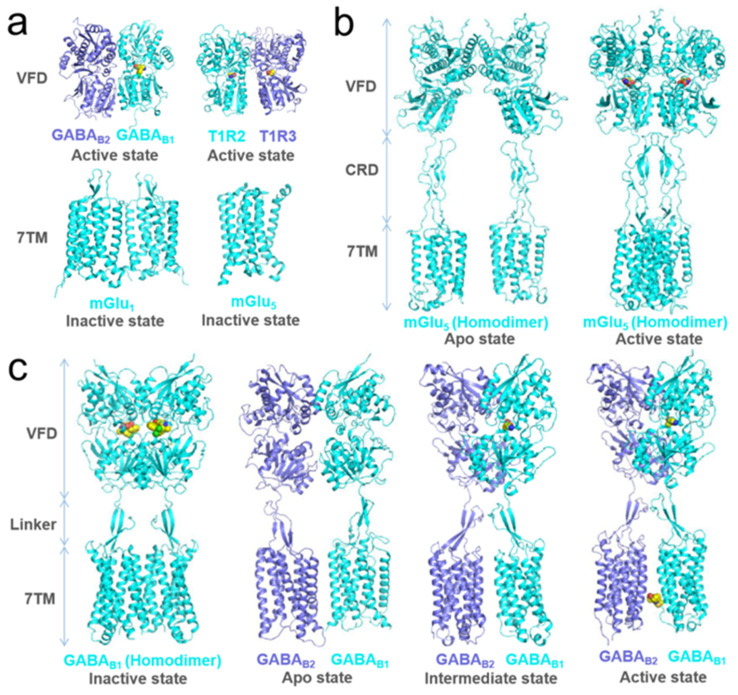
The solved structures of class C GPCR. (**a**) The VFD or 7TM structures of class C GPCR in active/inactive conformations: agonist-bound (active state) (PDB: 4 ms4) VFD structure of GABA_B2_ and GABA_B1_ heterodimer [37], agonist-bound (active state) (PDB: 5 x2 n) VFD structure of T1R2 and T1R3 heterodimer [38], 7TM structure (inactive state) (PDB: 4or2 and 4oo9) of mGlu_1_ [39] and mGlu_5_ [40]; The ligands are displayed as space-filling models and the heterodimer structures are indicated in blue and cyan, respectively. (**b**) The full-length structures of mGlu_5_ homodimer (cyan) [41] in different states apo (PDB: 6n52) and active (PDB: 6n51)); In the mGlu5 structures, VFD and 7TM domain are connected by a CRD (cysteine-rich domain) domain; Activation by two agonists leads to compaction of the mGlu5 dimer (right); The ligands are displayed as space-filling models and the homodimer structures are shown in cyan. (**c**) The full-length structures of GABA_B_ homodimer [42] or heterodimer [43] in different states inactive (PDB: 6w2y), apo (PDB: 6vjm), intermediate (PDB: 6uo9), and active (PDB: 6uo8)); In the GABA_B_ structures, VFD domain and 7TM domain are connected by a linker instead of a traditional CRD domain. GABA_B_ forms a very tight homodimer (inactive state) when bound with two antagonists and compact heterodimer (intermediate state) when bound with an agonist in the VFD domain, while GABA_B_ forms an incompact heterodimer in the apo state (the apo form and holo form are relative, the former refers to the protein structure that is not bound to the orthosteric molecule, and the latter refers to the bound structure). Furthermore, when bound with a positive allosteric modulator in the 7TM domain, as well as an agonist in the VFD domain, GABA_B_ is fully activated in the form of a compact heterodimer.

Class C GPCRs have a sizeable N-terminal domain, and almost all carry a bi-lobal VFD, homologous to the periplasmic bacterial proteins that bind amino acids and ions, and a cysteine-rich domain between the VFD and 7TM (also known as heptahelical domain) (Figure 2). The orthosteric ligands of the receptors bind between the lobes of the VFD domains, inducing the closed conformation of the VFD. When the 7TM domain of class C GPCR without the extracellular N-terminus is expressed, it could still be activated by positive allosteric modulators binding within it as class A GPCRs are activated by their orthosteric agonists [32,33]. Some class C receptors are homodimers (e.g., calcium-sensing receptor and metabotropic glutamate receptors (mGluRs)), whereas others are heterodimers consisting of two different protomers (GABAB receptors [34], sweet and umami taste receptors; mGluRs can also form heterodimers within subfamilies [35,36]). Interestingly, only one VFD from one protomer of the GABAB receptor binds the ligand, whilst the 7TM of the other protomer couples to a G protein [34], suggesting that the allosteric interactions between VFD and 7TM domains are necessary for receptor activation [5]. Inter-domain allosteric interactions and functional asymmetry, where only one protomer couples the G protein, appear common to all class C receptors. In homodimeric mGluRs, the agonist binding one VFD can activate this receptor, while binding both VFDs can further enhance the activity [5].

**Figure 3:**
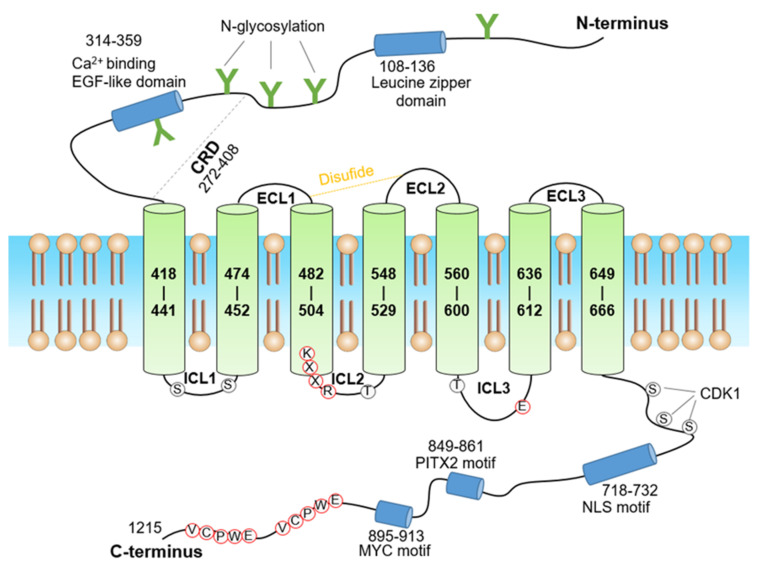
The schematic representation of human GPR158. The green letter “Y” indicates putative N-glycosylation sites in the N terminus. The disulfide bond in ECL1 and ECL2 is shown as a dotted yellow line. The leucine zipper domain, Ca2^+^-binding EGF-like domain, NLS motif, MYC motif, and PITX2 motif, are shown as blue cylinders. The conserved VCPWE motifs in the C-terminal tail of GPR158 are shown as golden hexagons. The KXXR motif in TM3 and the residue E in ICL3, presumed to be involved in the activation of Family C GPCRs, are marked in red circles. Three serine in the C-terminal domain, the putative phosphorylation sites for CDK1, may involve in cell proliferation. The figure was modified from: GPR158, an orphan member of G protein-coupled receptor Family C: glucocorticoid stimulated expression and novel nuclear role. Patel N, et al. PLoS One. 2013 [25]; used with permission from the publisher.

Since GPR158 was discovered, its functional research in the nervous system has been inseparable from RGS7. Patil, et al. reported the structures of the human GPR158 alone and bound with RGS7-Gβ5 employing single-particle cryogenic electron microscopy (cryoEM) recently [44]. Jeong, E., et al. also reported the cryo-EM structures of GPR158 alone and with RGS7-Gβ5 [45]. The structures of GPR158 stabilized by a pair of phospholipids and the complex of one RGS7–Gβ5 heterodimer were at an average resolution of 3.4 Å and 3.3 Å, respectively [44]. The overall architecture of GPR158 is composed of three parts: (1) a large N-terminus containing a signal peptide (AA 1-23) and multiple potential N-glycosylation sites (AA 98, 143, 215 and 274), (2) a canonical 7TM with three extracellular loops (ECL1-3) at the extracellular side intermingled with three intracellular loops (ICL1-3) at the intracellular side, and (3) an extended C-terminal region [46] (Figure 3).

The N-terminal portion of the extracellular domain (ECD) of GPR158 adopts a previously unrecognized characteristic Cache (calcium channels and chemotaxis receptors) domain in GPCRs [44], in which the Leucine zipper domains could recognize a specific DNA sequence and mediate dimerization [47]. The EGF-like calcium-binding domain may be crucial for numerous protein–protein interactions. The cysteine-rich domain (CRD) plays a role in receptor activation [41]. ECL2 C573 forms a conservative disulfide bond with TM3 C481, which is a conserved interaction conserved throughout many other GPCRs, and the disulfide bonds are essential for ligand recognition [44,48,49].

In the intracellular domain of GPR158, the KXXR motif in TM3 and the residue E (AA 609) in ICL3 are presumed to be involved in the activation of Family C GPCRs [28]. The bipartite nuclear-localization-signal (NLS) motif located in the C-terminus of GPR158, LKKLY and KRKK is essential for the nuclear entry [28]. Specific sequences for binding of transcription factors, such as c-Myc and Pitx2, are also found in the C-terminus of GPR158 [28]. As is known, MYC controls cell proliferation, while PITX2 is involved in the regulation of cell differentiation and organ development [50,51,52,53]. Upon translocation to the nucleus, it is likely that GPR158 interacts with these transcription factors. The two conserved VCPWE motifs in the GPR158 C-terminal tail regulate the interactions between GPR158 and G_o_ [26]. It is worth noting that GPR158 exhibits constitutive activity for Gi/o proteins, but not for Gq [54]. Interestingly, GPR158 localizes RGS7–Gβ5 and the activated Gαi/o protein [26,55]. Three serines in the C-terminal extracellular domain of GPR158 are the putative phosphorylation sites for protein kinases involving cell proliferation, such as CDK1 [28,56]. Taken together, the structural information for GPR158 provides insights into the unusual biology of the orphan receptors and the noncanonical signaling mechanism by which GRP158 selectively recruits the RGS7–Gβ5 complex.

## 4. Roles of GPR158 in Cancer

GPR158 is highly expressed in the brain and its expression shown to be specific in nervous system-related tumors [17]. Recent studies have shown that GPR158 may quantitatively characterize the malignant process of glioma (i.e., GPR158 expression was highest in the central nervous system (CNS) and oligodendrogliomas, lower in IDH mutant astrocytomas and lowest in the most malignant form of glioma and IDH wild-type glioblastoma) [17]. Remarkably, GPR158 may switch the glioma phenotypic plasticity via the downregulation of proliferation, migration and glioma stem-like cell formation, and via the induction of proneural differentiation and apoptosis simultaneously [17]. The neural differentiation of stem and progenitor cells is associated with apoptotic cell death [57]. The correlation of GPR158 expression with molecular subtypes, patient survival and therapy response suggest a possible role for GPR158 as a prognostic biomarker and a therapeutic target in human gliomas. It is worth noting that there was also some transcriptomic evidence of a common pathogenesis pathway in MDD (Major Depressive Disorder) and GBM [58]. In keeping with these findings, GPR158 becomes hypermethylated with the decreased expression of the invasive melanoma cells, which may affect the neural crest differentiation pathway and the regulation of the actin cytoskeleton [59]. GPR158 is also hypermethylated in many esophageal squamous cell carcinoma (ESCC) samples and can be used as a risk factor marker [60].

**Figure 4:**
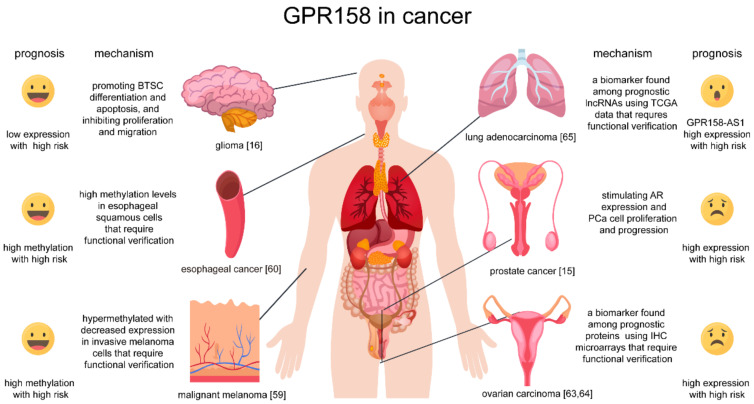
Roles of GPR158 in cancer. Current reports on the role of GPR158 in cancer. GPR158 may play different roles in different cancers. Smile icons indicate that high expression of GPR158 has a favorable prognosis, while sadness icons for high expression of GPR158 with a poor prognosis, and the surprise icon on the prognostic correlation for further verification. IHC, immunohistochemistry; BTSC, brain tumor stem cells; TCGA, The Cancer Genome Atlas. The figure was designed using resources from Freepik.com.

GPR158 expression is elevated in several cancer types. As mentioned, GPR158 expression was stimulated by androgens and promotes prostate cancer (PC) cell proliferation significantly [16]. A neuroendocrine tumor (NET) is a rare type of tumor that arises from specialized body cells mostly residing in the digestive tract [61,62]. GPR158 expression correlates with a neuroendocrine differentiation phenotype and promotes anchorage-independent colony formation implying a role for GPR158 in tumor formation [16]. GPR158 was also shown to be a histotype-specific prognostic biomarker in mucinous (MC) ovarian carcinomas, with elevated GPR158 expression patterns indicating unfavorable overall survival [63,64]. The involvement of GPR158 in the aforementioned aggressive clinical behavior and the subsequent poorer survival indicates that the GPR158-expressing neuroendocrine cells may represent transdifferentiated epithelial cells. Elevated lncRNA GPR158-AS1 (GPR158 Antisense RNA 1) expression was associated with poor patient outcome for lung adenocarcinoma (LUAD) [65], whereas the expression level of GPR158 AS1 was positively associated with GPR158 mRNA level [17]. Due to the role of lncRNA regulation at the transcription level and its aberrant expression patterns in various cancer types, the underlying molecular mechanisms of GPR158 and GPR158-AS1 still need to be explored. We summarized the different roles of GPR158 in cancer in Figure 4.

Further reports in the literature may highlight a causative role of GPR158 in human health and disease [66]. We list some as follows:

(1) Deregulating cellular metabolism: An mRNA microarray study on the subnuclear structures of the mouse brain suggested that habenular GPR158 might be involved in food consumption and energy expenditure (EE) [67]. Single nucleotide polymorphisms (SNPs) of GPR158 were found to be associated with a lower energy expenditure (EE) and adiposity in Native Americans [68]. As a known intracellular interacting protein of GPR158, RGS7 is at an obesity locus in humans [69] and as a putative agonist of GPR158, OCN mediates insulin signals in glucose metabolism [70], which indicated that GPR158 might influence tumor development and neuropsychiatric diseases through energy metabolism.
(2) Avoiding immune destruction: SNPs of the GPR158 gene were shown to be potentially liked to humoral immunity to smallpox vaccination [71] and to hepatitis C virus (HCV) clearance in patients of European and African ancestry [72]. These findings expand the relationship existing between GPR158 and neuronal activity towards its possible role in neuro-immune cross-talk.
(3) Senescence: An array of works from the literature reported that GPR158 was related to ageing cardiac disease [73], age-related memory loss [74] and Parkinson’s disease [75], which suggests GPR158 could act as an age-related marker.

Studies have been carried out to uncover the molecular mechanisms of GPR158 in the vast complexity of cancer phenotypes and genotypes. Fini investigated glucocorticoid (GC)-induced ocular hypertension (OH) [28]. Genecard expression data suggested that the mRNA is widely expressed in normal human tissues, while the protein is expressed only in the retina and prefrontal cortex. In keeping, the GPR158 protein expression was not detected until treated with the glucocorticoids dexamethasone (Dex) for six days or triamcinolone acetonide (TA) for eight days in cultured trabecular meshwork (TBM) cells [28]. GPR158 overexpression was linked to ocular hypertension, associated with TP53 pathway activation and enhanced cyclic adenosine 3′,5′-monophosphate (cAMP) production in response to epinephrine [76]. Of note, significantly higher levels of cAMP were nonetheless found in the medial prefrontal cortex (mPFC) of the *Gpr158^−/−^* mice [18]. Taken together, GPR158 may develop into a uniquely effective drug target for ocular hypertension and glaucoma [46] in the future. It was also found that GPR158 can interact with androgen receptors to modulate tumor cell proliferation via lowering the responding threshold to androgen during androgen deprivation therapy [16]. To date, GPR158 presents an intriguing target for prevention and therapy of castration-resistant prostate cancer (CRPC) [77].

We identified a target-dependent effect of microRNA-449a in inhibiting cell growth and migration by the downregulation of CCND1 and in suppressing neural phenotypes by the downregulation of GPR158 [17]. GPR158 can regulate the malignant phenotype of glioma, and, as a biomarker, quantitatively characterize the malignant process of glioma independent from the miR-449a target CCND1 (the expression of CCND1 remains largely independent of the tumor subtype [17]). Strikingly, GPR158 with a mutated NLS was internalized in small endocytic vesicles and retained in the cytoplasm, while the treatment of an inhibitor of endocytosis resulted in GPR158 trafficking to the plasma membrane. Failing to fulfil its functions in the nucleus, membrane GPR158 was not able to enhance cell proliferation [28]. The subcellular location of GPR158 may explain why it plays a different role in nervous system and somatic tumors.

## 5. Roles of GPR158 in Affective Disorders

GPR158 is extensively expressed in CNS, particularly in the prefrontal cortex (PFC), striatum and hippocampus, where it controls synapse formation and function [18,24,29,78]. We summarized the roles of GPR158 in affective disorders in Table 1. GPR158 has been demonstrated to be implicated in the etiology of affective disorders, for instance, memory loss, cognitive diseases and depression [18,19,39,74], mainly attributed to the fact that GPR158 plays a critical role in the structural organization and functional formation of the synapse. It was confirmed that a germline knockout (KO) of GPR158 could result in the interrupted dendritic structures of CA1 and CA3 circuits in the mouse hippocampus [29,78]. GPR158 deletion undermines the bouton morphology and ultrastructural organization of the active zone, decreases the density of postsynaptic terminals, augments the density of mossy fiber synapses and reduces synaptic transmission without affecting the adjacent inputs on the same dendrite [24,29].

GPR158 plays a part in mediating chronic stress-induced depression. A global abolishment of GPR158 led to an anti-depressive phenotype in mice, characterized by a lower susceptibility to learned helplessness and reduced anhedonia [18,19]. The rats exposed to chronic unpredictable stress (CUS) showed higher serum glucocorticoid (GC) level and anxiety-like behavior, but not depressive-like behavior, in the absence of GC production [79]. The persistent exposure to chronic stress could also enhance the expression level of GPR158 in mPFC in a GC-dependent fashion. GC increased the expression of GPR158 at the transcriptional and translational level [28,79,80], which suggests a role of the glucocorticoids–GPR158 axis in anxiety and depression. During the transition from a stressful state to depression, increased GPR158 expression induced by long-lasting stress resulted in RGS7 being directed to the plasma membrane, and this GPR158–RGS7 complex modulated the function of the GTPase accelerating protein (GAP) complex to regulate adenylate cyclase (AC) and cAMP production in the mPFC [19,24]. Further, Itakura, et al. found that GPR158 maintained the homeostasis of intraocular pressure, while GPR158 deficiency caused the inhibition of ageing-induced stress in the visual system [76]. These findings suggest new avenues for pharmacological interventions in affective disorders.

Of interest, postsynaptic GPR158 can bind cell surface glypican 4 (GPC4) to form synapse-organizing protein complexes, which can induce presynaptic differentiation and selectively mediate the formation of synaptic architecture and the function of mossy fiber-CA3 synapses [29]. GPR158 could mediate OCN’s regulation of hippocampal-dependent memory [31]. Furthermore, GPR158 ameliorates age-dependent memory loss mediated by the histone-binding protein RbAp48 through OCN/GPR158 signaling [74]. It is interesting that embryonic osteocalcin could regulate postnatal adrenal steroid through the GPR158 receptor [81]. This finding postulated translational potential as to whether modulating osteocalcin levels could promote endogenous adrenocortical function in adrenocortical hypoplasia and glucocorticoid deficiency [80]. Of importance, GPR158 appears to be correlated with ageing and cardiac diseases caused by aFGF (acidic fibroblast growth factor)-induced collagen deposition [73] and associated with the atrophy pattern [82] and Alzheimer’s Disease [83], which may be indicative for GPR158 in age-onset diseases [84]. There are data showing that GPR158 signaling could affect the brain-derived neurotrophic factor (BDNF) via the protein RbAp48 in the hippocampus and mPFC [74]. Fibroblast growth factor (FGF) may also influence GPR158 expression and could play a role in the differentiation of cardiac fibroblasts [73,85].

## 6. Discussion and Conclusions

**Figure 5:**
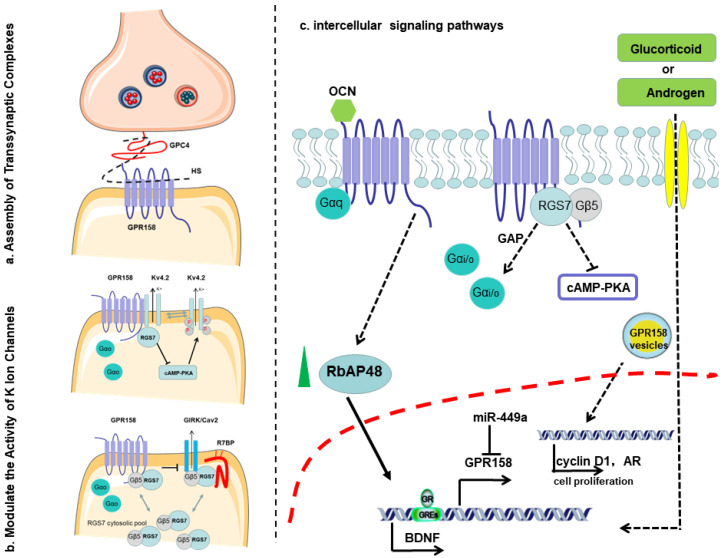
Roles of GPR158 in synaptic organization, ion permeability and signaling pathway mediation. (**a**) Presynaptic GPR158 can interact with presynaptic GPC4 to form a GPR158–GPC4 complex and organize the formation of synapse. (**b**) GPR158 can recruit RGS7 to form a GPR158–RGS7 complex, regulate cAMP concentration and further modulate the K^+^ and Ca^2+^ ion channel. (**c**) OCN are proposed to activate GPR158 and increase the RbAP48 level, thus affect BDNF and GPR158 level, while some hormones, including glucorticoid and androgen, can affect the transcription of GPR158. OCN, osteocalcin; GPC4, glypican 4; RGS7, regulator of G protein signaling 7; cAMP, cyclic adenosine monophosphate. Arrows indicate increased expression and blunt head means suppression.

We summarized the role of GPR158 in synaptic organization, ion permeability and signaling pathway mediation from an extracellular, membrane and intercellular perspective in Figure 5.

GPR158 features a large extracellular N-terminus that is commonly observed in adhesion GPCRs obtaining an EGF-like Ca^2+^ binding domain and a leucine zipper domain, which differs from typical class C GPCRs [24]. The N-terminus of GPR158 may bind to extracellular molecules, say, putative ligands. Recently, GPR158 were found to interact with GPC4 in trans to induce presynaptic differentiation and regulate the spine density in an input-specific manner [29] (Figure 5a). GPC4, as a glycosylphosphatidylinositol (GPI)-anchored HSPG, forms transsynaptic complexes with a variety of adhesion molecules (e.g., Leucine Rich Repeat Transmembrane Neuronal 4 (LRRTM4)) to mediate excitatory synapse formation [86]. Notably, the paralogue of GPR158, GPR179, also interacts with the HSPG pikachurin via ectodomain [30]. In the hippocampus, GPR158 may be activated by interaction with OCN [31]. OCN is a multipurpose bone-derived hormone and is necessary for hippocampal-dependent memory and to reduce anxiety-like behavior [87,88]. OCN has a small molecular weight, only 46–55 amino acids, which allows it to cross the blood–brain barrier and to bind to neurons in specific brain regions. OCN subsequently triggered BDNF signaling through GPR158, constituting a molecular pathway critical for hippocampal-dependent memory [31,89]. OCN could also increase RbAp48 in the dentate gyrus (DG) and then RbAp48 occupies the promoter region of BDNF and GPR158 for a transcriptional regulation [74]. Notably, BDNF expression is decreased with age [90]. OCN could regulate the glucose metabolism and therefore promotes neuronal survival through inhibiting pyroptosis [91]. GPR158 may be the main receptor of OCN to stimulate adrenal function given the observation that in the *Gpr158^−/−^* mouse model the corticosteroid levels were decreased and OCN injections failed to rescue the endocrine phenotype [81].

With the development of the integrated computational and multifaceted experimental approach, elucidating the peptide–GPCR network and even screening the putative ligands of GPR158 in silico are promising avenues of research [92]. Furthermore, the identification of trans-synaptic HSPG binding partners of GPR158 [29] provided a biochemical means for interrogating candidate ligands of GPR158, as well as its diversified biological roles in association with diseases.

The signals from and to the GPR158/RGS7 complex are central for understanding the biology of this receptor. GPR158 C-terminal domain is an essential modulator of RGS7 function and stabilization [26]. Being a potent negative modulator of Gi/o signaling of the Gα proteins, RGS7 binds to Gβ5, forming a dimer and suppressing G-protein function as a GTPase activating protein (GAP) [93]. The GPR158 recruitment of RGS7/Gβ5 to the plasma membrane accelerates the deactivation of Gi/o signaling [24,55]. On the other hand, GPR158 and GPR179 can simultaneously activate Gi/o [54]. GPR158 traps Gαo through the VCPWE motifs, possibly leading to its preclusion from interacting with βγ and prolong the βγ activity [26], in keeping with the finding that RGS7 promotes the dissociation of GPR158 from Gαo, suggesting that the binding of GPR158 to RGS7/Gβ5 may exert an opposite function as bound to Gαo alone [44]. Additionally, GPR158 may be activated by the interaction with OCN in the hippocampus to cascade signals via a Gαq [31].

cAMP, as a known second messenger for intracellular signal induction, is synthesized via adenosine triphosphate (ATP) from G-proteins upon the activation of GPCRs. cAMP production is controlled by a number of neurotransmitters through GPCRs signals towards Gs or Gi [94]. The recruitment of RGS7 via GPR158 in mPFC suppresses the homeostatic regulation of cAMP [19]. Eerily, higher levels of cAMP were found in *Gpr158*^−/−^ mice mPFC [18], whilst the presence of GPR158 also enhanced cAMP production in response to epinephrine in TM-1 (human trabecular meshwork) cell line [76]. The GPR158-RGS7 regulation of cAMP production could be completely abolished by the Gβγ-scavenger peptide [19].

A plethora of GPCRs, such as GABAB receptors (GABABRs), have been found to be engaged in membrane excitability and synaptic transmission in the brain [95]. The neurotransmitter γ-aminobutyric acid (GABA) initiates the synaptic inhibition activity of GABABRs coupling to G-proteins and ion channels, inwardly rectifying K^+^ (GIRK) and P/Q/N type voltage-gated Ca2^+^ (CaV2) ion channels [96]. GPR158 is implicated in such neuro-modulatory activities. RGS7 formed a complex with its binding protein R7BP, which prominently accelerated the kinetics of GIRK and CaV2 through GABABRs; however, when “clamped” with GPR158, it inhibited GIRK and CaV2 by GABA [96] (Figure 5b). Further, the GPR158-RGS7 signaling node modulated depression via physically interacting with the Kv4.2 channel and promoted its function by mediating the cAMP–protein kinase A (PKA) phosphorylation [97] (Figure 5c). Nonetheless, the explicit functional consequences of the GPR158-RGS7-G protein cascade remain to be elucidated.

The disrupted expression of GPR158 involves the etiology of many affective disorders, e.g., neurodegenerative diseases, memory loss and stress-induced depression, in line with the recent findings concluded from genome-wide associated studies (GWAS) and post-mortem transcriptional analyses [98]. That suggests new avenues for pharmacological interventions in affective disorders. Some hormones, including glucorticoids (GC) and androgens, can affect GPR158 expression at the transcription level. Firstly, GC treatment stimulated GPR158 mRNA expression through GC responsive elements (GREs) in the GPR158 5′-upstream promoter [28]. Newly synthesized GPR158 traffics to the plasma membrane, where it could be rapidly internalized in small endocytic vesicles for translocation to the nucleus [28]. Secondly, GPR158 expression could also be induced by androgens, but GPR158 promotes PCa cell proliferation independent of AR functionality, which requires its entry into the nucleus [16] (Figure 5c). The interruption of GPR158 expression could nonetheless influence the level of androgens, probably 5-HT, oxytocin, and ACTH in the plasma and BDNF in the brain, which contributes to the etiology of affective disorders and tumors [16,74,99,100].

This review presented recent findings regarding the evolutionary origin, sequences, structures and function in disease of GPR158, promoting it as a promising candidate drug target. The structure of the GPR158 homodimer bound to the RGS7–Gβ5 complex has shed light on how GPR158 may detect small molecule ligands with its Cache domain. Given that the RGS protein binds the same elements of G proteins and β-arrestins, through which GPCRs transduce signals, how ligand–GPR158 complexes are engaged in the activities of these downstream effectors will be the focus of future studies and promises to yield many benefits. Beyond the structural implications, it is critical to consider that extracellular and intracellular interaction partners that can endow the GPCR to elicit specific functional consequences. The characterization of GPR158 is a promising dimension in GPCR function and pharmacology. To this end, GPR158 may serve as a bridge for revealing the relationship between cancer and neuroscience and for developing potential new treatments.

## Figures and Tables

**Table 1:** Roles of GPR158 in affective disorders.

Disorders/Phenotypes	Results of Studies
Human studies:	
Major Depressive Disorder	↑ GPR158 in dlPFC [18,19]
Animal studies:	
Stress-induced Depression	↑ GPR158 in mPFC, under chronic PRS [18]
	↑ GPR158 in mPFC, under UCMS [18]
	↑ GPR158 in mPFC, with chronic corticosterone treatment [18]
	↑ GPR158 in primary cortical neurons, with chronic corticosterone treatment [18]
	GPR158 OE in mPFC↑ Immobility in FST [18]
	GPR158 KO ↓ Immobility in FST [18]
	GPR158 KO↓ Marble buried in MBT [18]
	GPR158 KO↑ Time in open arms in EMP, 2–4 month olds [18]
	GPR158 KO ↓ time in open arms in EMP, 3 month-old females [18]
	GPR158 KO ↓ time in lit box in LDT, 3 month-old females [31]
	GPR158 KO↓ time in center in OFT, 3 month-old females [31]
	GPR158 KO ↔ time in center in OFT, 8–12 week-old males [78]
	GPR158 KO ↔ immobility time after yohimbine injection in FST, 2–4 month-old males [19]
	GPR158 KO↓ immobility in TST, 2–4 month olds [18]
	GPR158 OE in mPFC↑ immobility in TST, 2–4 month olds [18]
	GPR158 KO ↔ immobility time in TST, after yohimbine injection (not after vehicle injection),2–4 month-old males [19]
Age-related Memory Loss	Disrupted GPR158/OCN signaling in the hippocampus [74]
Impaired Spatial Learning	Disrupted CA1 morphology and impaired spatial memory acquisition, GPR158 global KO [55,78]
Presynaptic Differentiation	↑ Mossy fiber synapse density, impaired postsynaptic density and synaptic strength, GPR158 global KO [29]

dlPFC, dorsolateral prefrontal cortex; mPFC, medial prefrontal cortex; PRS, physical restraint stress; UCMS, unpredictable chronic mild stress; FST, forced swim test; MBT, marble burying test; EPM, elevated plus maze; LDT, light–dark transition; OFT, open field test; TST, tail suspension test.
